# A water-soluble supramolecular complex that mimics the heme/copper hetero-binuclear site of cytochrome *c* oxidase†
†This paper is dedicated to (late) Professor Takashi Ogura.
‡
‡Electronic supplementary information (ESI) available. See DOI: 10.1039/c7sc04732k


**DOI:** 10.1039/c7sc04732k

**Published:** 2018-01-15

**Authors:** Hiroaki Kitagishi, Daiki Shimoji, Takehiro Ohta, Ryo Kamiya, Yasuhiro Kudo, Akira Onoda, Takashi Hayashi, Jean Weiss, Jennifer A. Wytko, Koji Kano

**Affiliations:** a Department of Molecular Chemistry and Biochemistry , Faculty of Science and Engineering , Doshisha University , Kyotanabe , Kyoto 610-0321 , Japan . Email: hkitagis@mail.doshisha.ac.jp; b Picobiology Institute , Graduate School of Life Science , University of Hyogo , RSC-UH LP Center , Hyogo 679-5148 , Japan; c Department of Applied Chemistry , Graduate School of Engineering , Osaka University , 2-1 Yamadaoka , Suita 565-0871 , Japan; d Institut de Chimie de Strasbourg , UMR 7177 , CNRS , Université de Strasbourg , 4 Rue Blaise Pascal , 67000 Strasbourg , France

## Abstract

In mitochondria, cytochrome *c* oxidase (C*c*O) catalyses the reduction of oxygen (O_2_) to water by using a heme/copper hetero-binuclear active site. Here we report a highly efficient supramolecular approach for the construction of a water-soluble biomimetic model for the active site of C*c*O. A tridentate copper(ii) complex was fixed onto 5,10,15,20-tetrakis(4-sulfonatophenyl)porphinatoiron(iii) (Fe^III^TPPS) through supramolecular complexation between Fe^III^TPPS and a per-*O*-methylated β-cyclodextrin dimer linked by a (2,2′:6′,2′′-terpyridyl)copper(ii) complex (Cu^II^TerpyCD_2_). The reduced Fe^II^TPPS/Cu^I^TerpyCD_2_ complex reacted with O_2_ in an aqueous solution at pH 7 and 25 °C to form a superoxo-type Fe^III^–O_2_^–^/Cu^I^ complex in a manner similar to C*c*O. The pH-dependent autoxidation of the O_2_ complex suggests that water molecules gathered at the distal Cu site are possibly involved in the Fe^III^–O_2_^–^/Cu^I^ superoxo complex in an aqueous solution. Electrochemical analysis using a rotating disk electrode demonstrated the role of the FeTPPS/CuTerpyCD_2_ hetero-binuclear structure in the catalytic O_2_ reduction reaction.

## Abstract 2

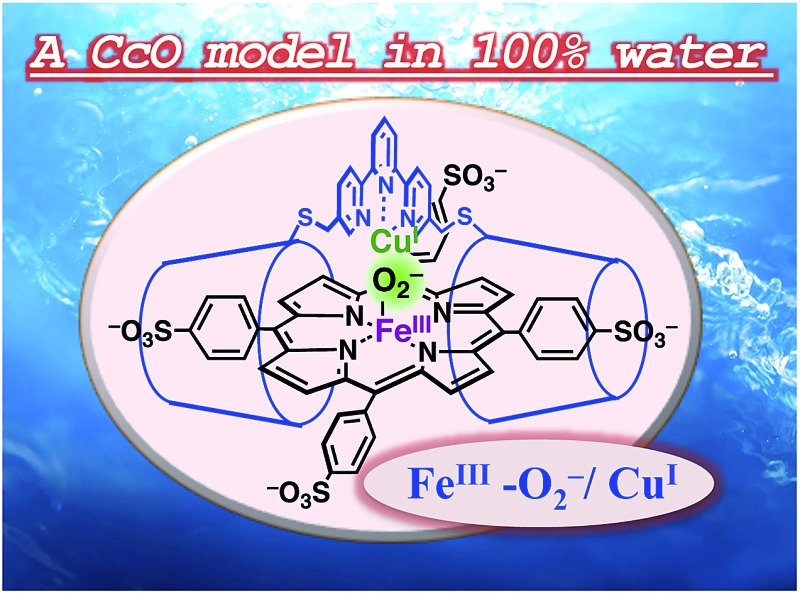
The O_2_ adduct of an aqueous synthetic heme/copper model system built on a porphyrin/cyclodextrin supramolecular complex has been characterized.

## Introduction

**Fig. 1:**
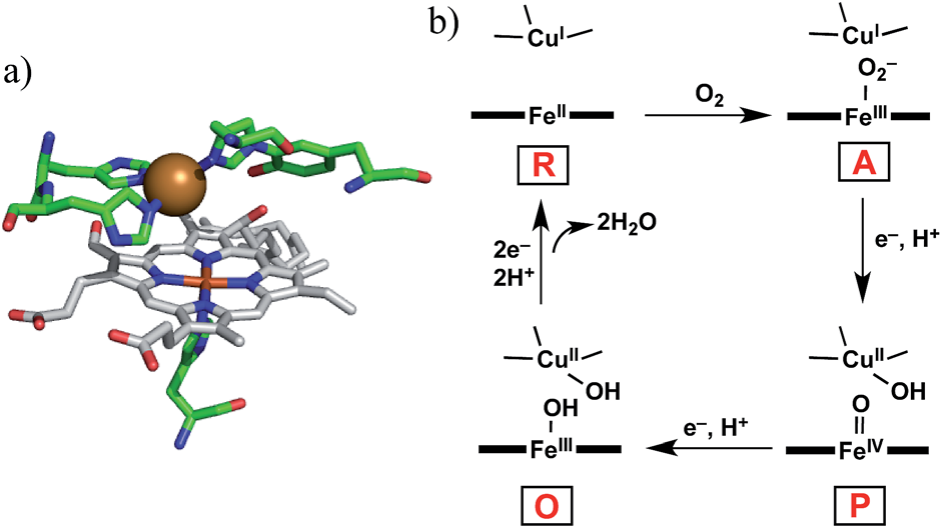
(a) Heme *a*_3_/Cu_B_ hetero-binuclear active site of C*c*O (PDB ID: ; 1OCO) and (b) the simplified mechanism for the O_2_ reduction reaction catalysed by C*c*O.

Cytochrome *c* oxidase (C*c*O) is the terminal enzyme in the mitochondrial respiratory chain. C*c*O consumes most of the molecular oxygen (O_2_) processed by living organisms by reducing it to water (H_2_O).^1^ The four-electron/four-proton reduction process (O_2_ + 4e^–^ + 4H^+^ → 2H_2_O) takes place at the heme *a*_3_/Cu_B_ hetero-binuclear active centre of C*c*O (Fig. 1a).^1^–^5^ For the catalytic O_2_ reduction reaction, the reaction mechanism schematically depicted in Fig. 1b has been proposed.^1^–^3^ In the catalytic cycle, the fully reduced heme *a*_3_/Cu_B_ site (Fe^II^/Cu^I^, compound R) reacts with O_2_ to form an oxymyoglobin-like superoxo complex of heme *a*_3_ (Fe^III^–O_2_^–^/Cu^I^, compound A).^3^,^6^ Compound A is rapidly (∼0.5 ms) converted to an oxoferryl intermediate (Fe^IV^=O/Cu^II^–OH, compound P) *via* O–O bond cleavage assisted by H atom injection from a vicinal tyrosine residue.^3^–^6^ Mechanistic investigations have suggested that one or more water molecules near the bound O_2_ can facilitate the conversion of compound A to compound P.^7^,^8^

To understand the reaction mechanism, synthetic heme/copper models have been constructed using tetraarylporphinatoiron(ii) (PFe^II^) combined with Cu^I^ complexes (Cu^I^L_*n*_, where L is a nitrogen donor ligand; *n* (coordination number) = 3 or 4).^4^,^5^ However, upon oxygenation of the PFe^II^/Cu^I^L_*n*_ model systems in anhydrous organic solvents, μ-peroxo-type bridged structures, *i.e.*, PFe^III^–O_2_–Cu^II^L_*n*_ complexes, tend to form instead of compound A-like superoxo species.^9^–^12^ In native C*c*O, the μ-peroxo-type bridged structure has not been experimentally identified, although it has been proposed as a transitional precursor of compound P.^3^,^12^,^13^ The structural differences between the native and model systems (superoxo *vs.* μ-peroxo)^14^ might be attributed to the influence of water.^7^,^8^,^13^ A model study by Naruta and co-workers demonstrated that the μ-peroxo complex (PFe^III^–O_2_–Cu^II^L_3_) formed at –70 °C was converted to the superoxo complex (PFe^III^–O_2_^–^/Cu^I^L_3_) at –30 °C by the action of water molecules.^15^ In native C*c*O, highly ordered water molecules have been detected in the vicinity of heme *a*_3_/Cu_B_.^7^,^16^ A quantum chemical calculation suggested that a water molecule in the vicinity of Cu_B_ decreases the energy barrier of the transformation of compound A to compound P.^8^ In this context, a water-soluble PFe^II^/Cu^I^L_*n*_ model compound would be useful to investigate the role of water on the reactivity of the Fe/Cu hetero-binuclear complex with O_2_. However, very few heme/copper mimics functioning under aqueous conditions have been prepared so far, except for the system constructed in the engineered heme pocket of myoglobin.^17^,^18^

**Scheme 1:**
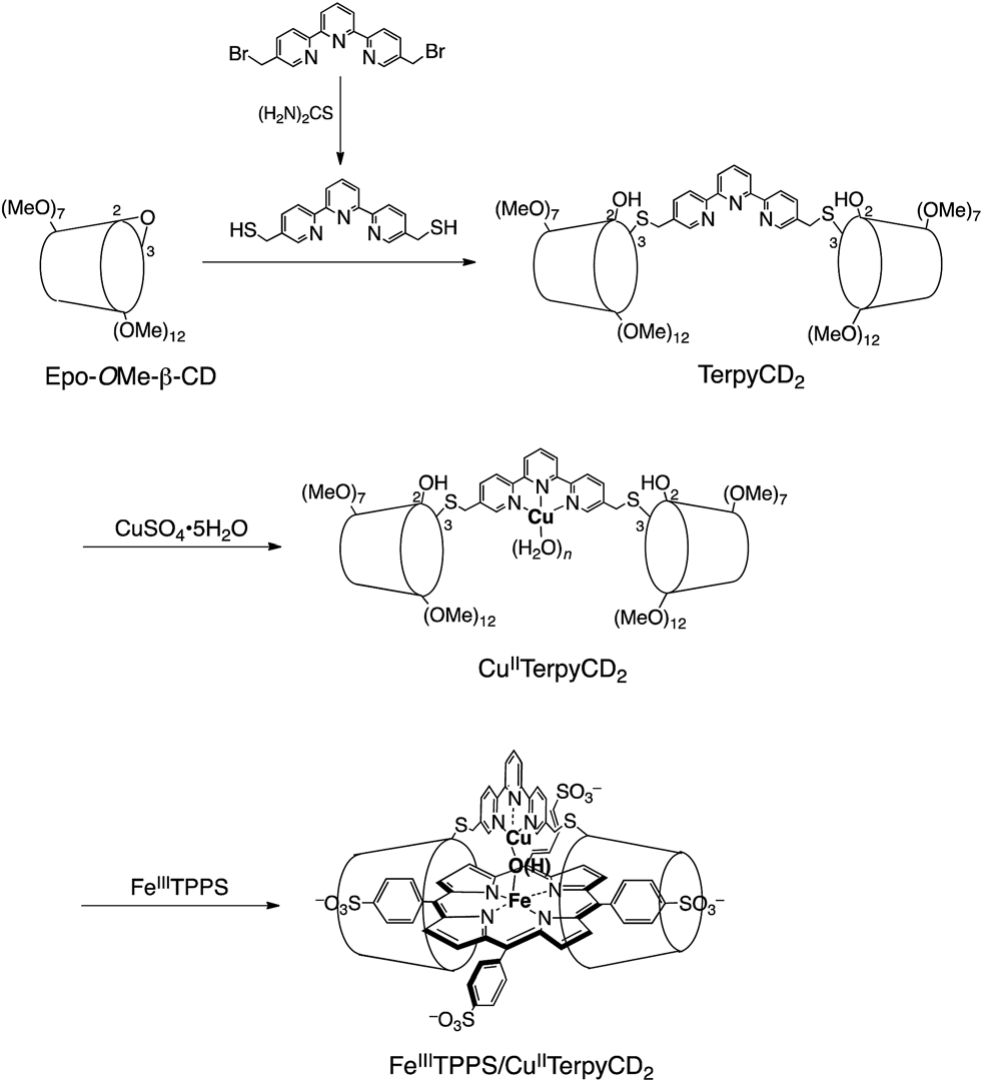
Preparation of the supramolecular Fe^III^TPPS/Cu^II^TerpyCD_2_ complex.

In this study, we describe an aqueous synthetic PFe/CuL_3_ hetero-binuclear model system built on a porphyrin/cyclodextrin supramolecular complex (Scheme 1). This system takes advantage of the very stable formation of a self-assembling 1 : 2 complex of 5,10,15,20-tetrakis(4-sulfonatophenyl)porphinatoiron (FeTPPS) with per-*O*-methylated β-cyclodextrins (CDs).^19^ We have previously studied the porphyrin/cyclodextrin complexes as simple biomimetic models of heme proteins that function under aqueous conditions,^20^–^23^ where the molecular cage of per-*O*-methylated β-CDs provided a microscopic hydrophobic environment for FeTPPS similar to the heme pocket of heme proteins.^24^ Here, we have synthesised a per-*O*-methylated β-CD dimer linked by a Cu^II^–terpyridine complex (Cu^II^TerpyCD_2_, Scheme 1) to replicate the distal tridentate Cu_B_ site of C*c*O. The structural characterisation of the supramolecular FeTPPS/CuTerpyCD_2_ complex and its reactivity towards O_2_ are described.

## Results and discussion

### Synthesis of a water-soluble Fe^III^/Cu^II^ hetero-binuclear complex

**Fig. 2:**
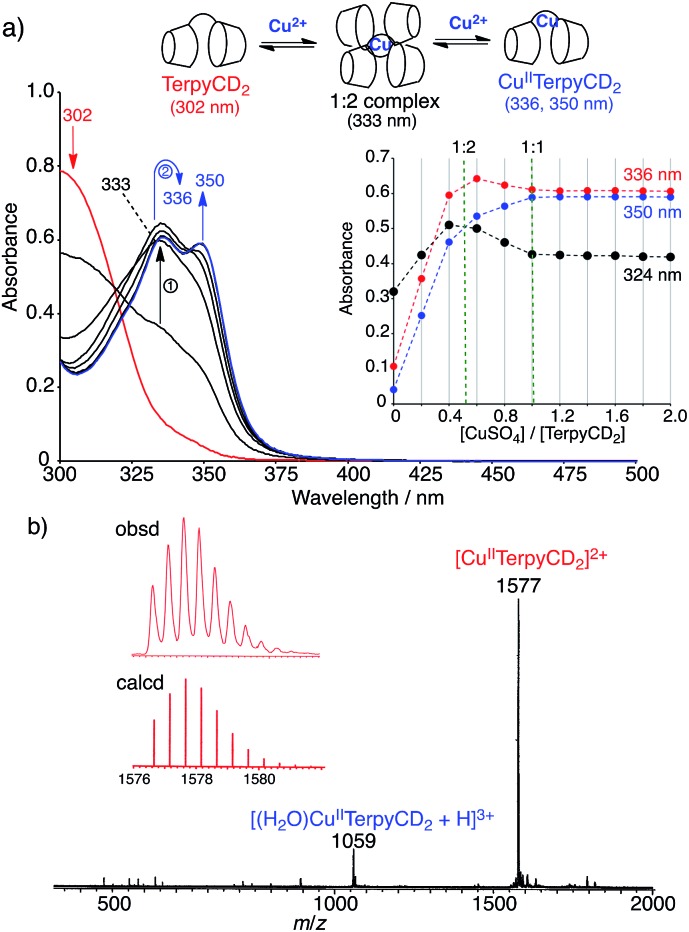
Complexation of TerpyCD_2_ with Cu^2+^ in aqueous solution. (a) UV-vis spectral change of TerpyCD_2_ (33 μM) upon stepwise addition of CuSO_4_ in water at 25 °C. The inset shows changes in absorbances as a function of [CuSO_4_]/[TerpyCD_2_]. The biphasic titration curve indicates transient formation of the 1 : 2 complex before forming the thermodynamically stable 1 : 1 complex (Cu^II^TerpyCD_2_) during the titration. (b) Electrospray mass spectrum (positive mode) of the 1 : 1 mixture of TerpyCD_2_ and CuSO_4_ in H_2_O. The inset shows the simulated isotope distribution patterns for the [Cu^II^TerpyCD_2_]^2+^ complex.

The synthetic route of a supramolecular Fe^III^TPPS/Cu^II^TerpyCD_2_ complex is shown in Scheme 1 and experimental details are described in (ESI‡). Briefly, the terpyridyl ligand was inserted as a linker of the CD dimer (TerpyCD_2_) by the reaction of 5,5′′-bis(mercaptomethyl)-2,2′:6′,2′′-terpyridine with 2,3-monoepoxy-per-*O*-methylated β-CD (Epo-*O*Me-β-CD).^20^ The addition of CuSO_4_·5H_2_O to TerpyCD_2_ in an aqueous solution generated two absorption bands at 336 and 350 nm (Fig. 2a), which corresponded to the ligand to metal charge transfer bands of the terpyridyl–Cu^II^ 1 : 1 complex.^25^ In the UV-vis titration, a biphasic spectral change was observed (Fig. 2a inset), indicating that the 1 : 2 complex of Cu^2+^ with TerpyCD_2_ (*λ*_max_ = 333 nm) was first formed and then it was converted to the thermodynamically stable 1 : 1 complex upon further addition of Cu^2+^. The spectral changes were completed at one equivalent of Cu^2+^. The complexation between TerpyCD_2_ and Cu^2+^ was also monitored by electrospray mass spectroscopy. In the 1 : 1 mixture of CuSO_4_ and TerpyCD_2_ in H_2_O, the 1 : 1 complex (Cu^II^TerpyCD_2_) was observed at *m*/*z* 1577 and 1059 (Fig. 2b), which corresponds to [Cu^II^TerpyCD_2_]^2+^ and [(H_2_O)Cu^II^TerpyCD_2_ + H]^3+^, respectively. The 1 : 2 complex was also detected as a small ion peak when the 1 : 2 mixture of CuSO_4_ and TerpyCD_2_ in H_2_O was analysed by electrospray mass spectroscopy (data not shown).

**Fig. 3:**
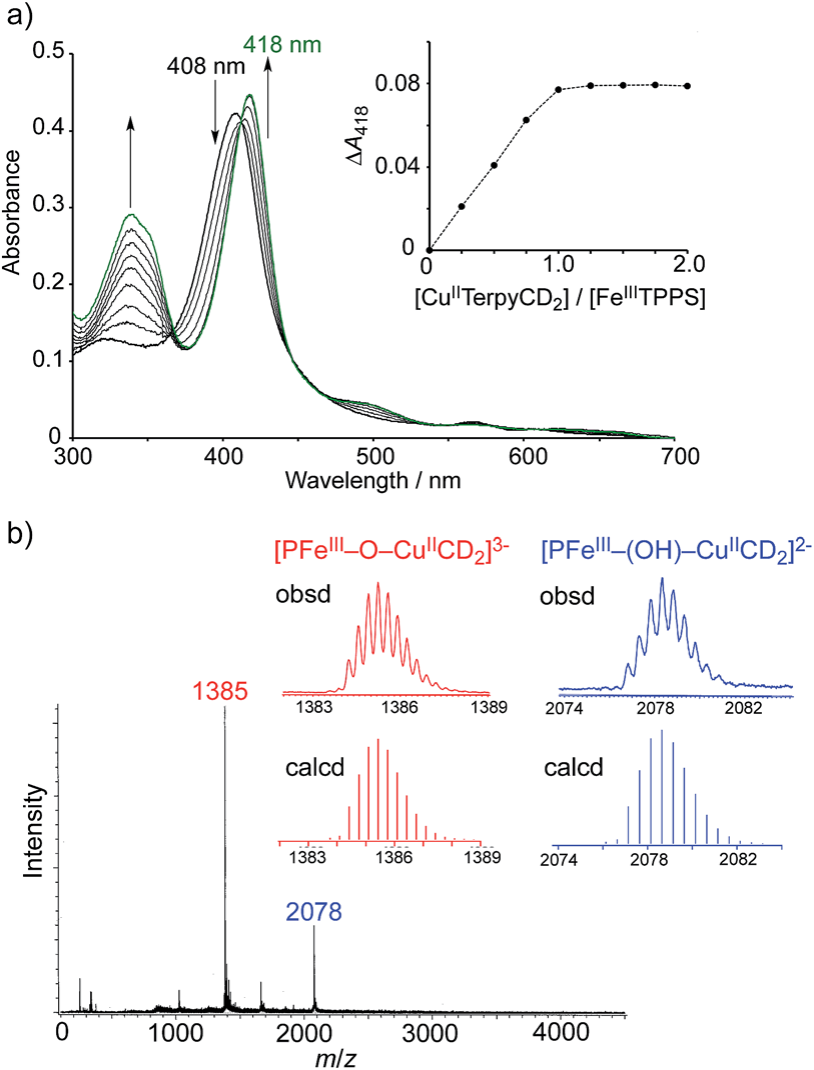
Characterisation of the supramolecular Fe^III^TPPS/Cu^II^TerpyCD_2_ complex. (a) UV-vis spectral changes of Fe^III^TPPS (3 μM) upon stepwise addition of Cu^II^TerpyCD_2_ in 0.05 M phosphate buffer at pH 7.0 and 25 °C. The inset shows the changes in absorbance at 418 nm as a function of the molar ratio ([Cu^II^TerpyCD_2_]/[Fe^III^TPPS]). (b) Electrospray mass spectrum (negative mode) of the 1 : 1 mixture of Fe^III^TPPS and Cu^II^TerPyCD_2_ in H_2_O. The inset shows the simulated isotope distribution patterns for the μ-oxo- and μ-hydroxo-bridged Fe^III^TPPS/Cu^II^TerpyCD_2_ complexes.

**Fig. 4:**
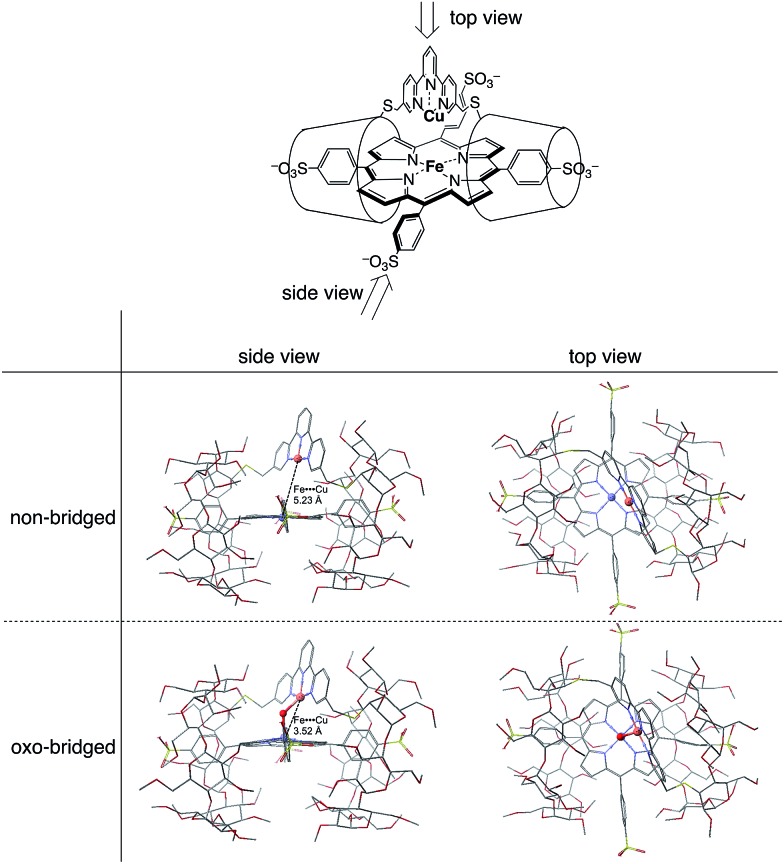
Optimized molecular structures of the FeTPPS/CuTerpyCD_2_ inclusion complexes in the Fe/Cu non-bridged and Fe/Cu oxo-bridged forms. The models are shown from both side and top views. Hydrogen atoms are omitted for clarity. Molecular mechanics calculations were carried out using CONFLEX/MM3 (extensive search) parameters in Scigress version 2.2.1 software program (Fujitsu).

The Cu^II^TerpyCD_2_ complex was then titrated with Fe^III^TPPS (Fig. 3a). The Soret band of Fe^III^TPPS shifted from 408 nm to 418 nm, indicating that a μ-oxo-dimer of Fe^III^TPPS dissociated to the monomeric monohydroxo complex (Fe^III^(OH^–^)TPPS)^19^ through interaction with Cu^II^TerpyCD_2_. The spectral changes were completed upon addition of one equivalent of Cu^II^TerpyCD_2_ to Fe^III^TPPS, indicating a quantitative 1 : 1 complexation. The obtained complex was then analysed by electrospray mass spectroscopy. The two main ion peaks were detected at *m*/*z* 1385 and 2078 as tri- and di-anionic species, respectively (Fig. 3b). Considering total charges of the complexes, the peaks at *m*/*z* 1385 and 2078 were assigned to the μ-oxo and μ-hydroxo Fe^III^TPPS/Cu^II^TerpyCD_2_ complexes, *i.e.*, [PFe^III^–O–Cu^II^CD_2_]^3–^ and [PFe^III^–(OH)–Cu^II^CD_2_]^2–^, respectively. The assignments were confirmed by isotope pattern simulations (Fig. 3b inset). Evidence of the μ-oxo (Fe^III^–O–Cu^II^) structure was also provided by its characteristic absorption bands at 453 and 567 nm, which appeared when the pH of the solution was increased (Fig. S3‡). The red-shifted Soret band at alkaline conditions indicates formation of the PFe^III^–O–Cu^II^ μ-oxo complex.^26^–^28^ The pH titration revealed the acid–base equilibrium of [PFe^III^–O–Cu^II^CD_2_]^3–^ and [PFe^III^–(OH)–Cu^II^CD_2_]^2–^ with p*K*_a_ = 8.8. This p*K*_a_ value is consistent with that previously predicted by Karlin and Blackburn (p*K*_a_ = 8 ± 2.5).^28^ The electron paramagnetic resonance (EPR) spectra showed significantly weak signals at *g* = 6.09 and 2.08 in the Fe^III^TPPS/Cu^II^TerpyCD_2_ complex (Fig. S4‡) because of the antiferromagnetic coupling between the two metal ions as a result of their close proximity. The optimized molecular structure (Fig. 4) also illustrates the proximity of Fe and Cu ions in the Fe^III^TPPS/Cu^II^TerpyCD_2_ complex; the Fe/Cu distances for the non-bridged and oxo-bridged forms are 5.23 and 3.52 Å, respectively. The distances are similar to those in native C*c*O, in which the oxidised heme *a*_3_/Cu_B_ distance were found in the range of 4.4–4.9 Å.^4^

### Characterisation of an O_2_ adduct of the Fe^II^/Cu^I^ complex

**Fig. 5:**
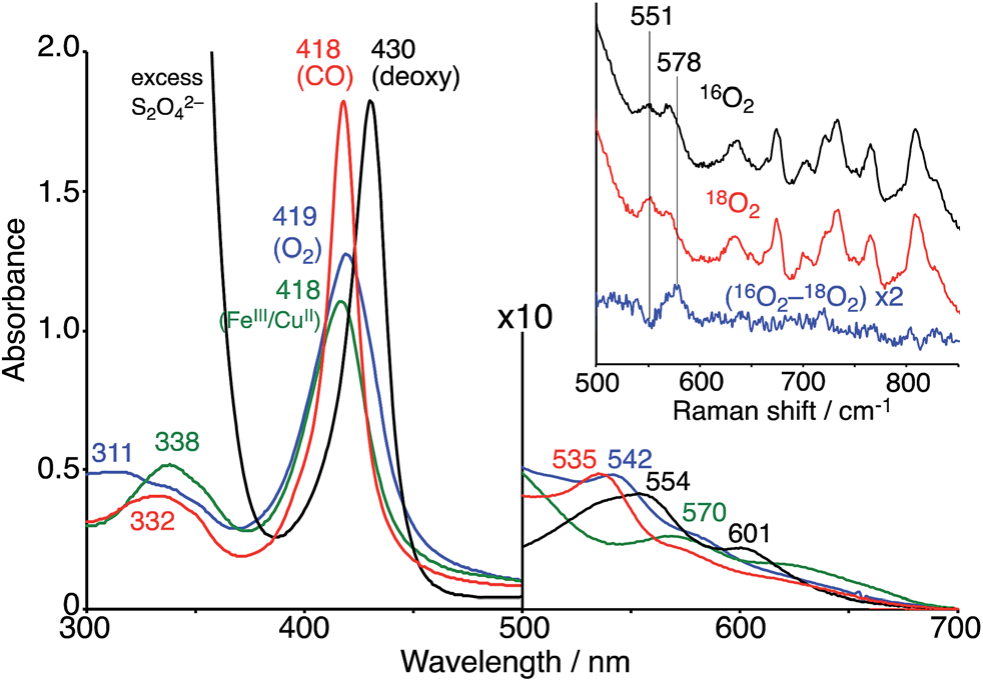
UV-vis spectra of the Fe^III^TPPS/Cu^II^TerpyCD_2_ (green) and its reduced Fe^II^TPPS/Cu^I^TerpyCD_2_ complexes in the deoxy (black), oxy (blue) and CO (red) forms in 0.05 M phosphate buffer at pH 7.0 and 25 °C. The inset shows the resonance Raman spectra of the Fe^II^TPPS/Cu^I^TerpyCD_2_ complexes obtained by excitation at 405 nm under ^16^O_2_ atmosphere (black), ^18^O_2_ atmosphere (red), and the difference ^16^O_2_–^18^O_2_ (blue). Conditions: 0.05 M phosphate buffer at pH 7.0, 77 K (frozen solution).

The Fe^III^TPPS/Cu^II^TerpyCD_2_ complex was reduced with excess sodium dithionite (Na_2_S_2_O_4_) to obtain the fully reduced [PFe^II^/Cu^I^CD_2_]^3–^ complex in the deoxy state in an O_2_-free solution (*λ*_max_ at 430, 554, and 601 nm, Fig. 5, black line). The dissolved O_2_ in the solution was completely consumed by excess dithionite, and the redox potential of dithionite is negative enough to reduce both Fe^III^ and Cu^II^ to Fe^II^ and Cu^I^.^29^,^30^ After the reduction, the solution was passed through a short gel-filtration column (Sephadex G-25) under aerobic conditions to remove excess S_2_O_4_^2–^ and its oxidised products. The UV-vis spectrum of the resulting solution showed absorption maxima at 419 nm and 542 nm (Fig. 5, blue line); the Q-band was very different from that of the oxidised state (Fe^III^TPPS/Cu^II^TerpyCD_2_, *λ*_max_ (Q-band) = 570 nm, green line) and similar to that of the O_2_ complex of the previously reported Fe^II^TPPS/CD dimer system.^20^ Introduction of CO gas into the solution caused further spectral changes with absorption maxima at 418 nm and 535 nm (Fig. 5, red line). The sharp Soret band is characteristic of the CO–Fe^II^TPPS complex,^20^ indicating that a ligand exchange from O_2_ to CO occurs in this system.

**Fig. 6:**
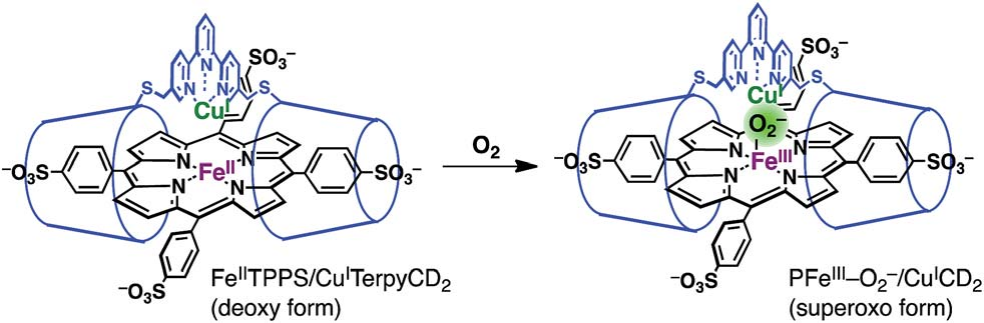
Oxygenation of the Fe^II^TPPS/Cu^I^TerpyCD_2_ complex to form a superoxo PFe^III^–O_2_^–^/Cu^I^CD_2_ complex.

The O_2_ complex was further characterized by EPR and resonance Raman (rR) spectroscopic analyses. The EPR spectrum of the O_2_ adduct of Fe^II^TPPS/Cu^I^TerpyCD_2_ measured at 77 K was completely silent (Fig. S4‡), which was consistent with the spectra of other O_2_ complexes of the PFe^II^/Cu^I^L_*n*_ hetero-binuclear systems.^31^–^33^ The rR analysis at 77 K (frozen solution of the O_2_ adduct) using 405 nm excitation revealed a characteristic band at 578 cm^–1^, which shifted to 551 cm^–1^ under an ^18^O_2_ atmosphere (Fig. 5 inset). The isotope shift (Δ*ν* = 27 cm^–1^) corresponds to the expected value for the *ν*_Fe–O_ stretching mode.^15^ The wavenumber is quite similar to those of the PFe^III^–O_2_^–^/Cu^I^L_*n*_ superoxo complexes in the previously reported native^34^ and synthetic model systems as listed in Table 1.^14^,^15^,^35^ Furthermore, the O–O bond stretching mode (*ν*_O–O_) was not enhanced in this system. This is a relevant observation as the *ν*_O–O_ band is often observed in the range of 750–900 cm^–1^ in the PFe^III^–O_2_–Cu^II^L_*n*_ μ-peroxo complexes, but not in the case of the Fe^III^–O_2_^–^/Cu^I^L_*n*_ superoxo complexes (Table 1).^14^,^15^,^35^–^37^ Based on the rR data, the configuration of the present O_2_-adduct of Fe^II^TPPS/Cu^I^TerpyCD_2_ is assigned as the superoxo-type PFe^III^–O_2_^–^/Cu^I^L_3_ complex (Fig. 6), which is the same coordination mode as in compound A of native C*c*O.^3^,^14^,^38^

**Table 1:** The Fe–O and O–O stretching frequencies (*ν*_Fe–O_/cm^–1^, *ν*_O–O_/cm^–1^) in the O_2_ complexes of native C*c*O and synthetic PFe/CuL_*n*_ compounds

	*ν*_Fe–O_/cm^–1 16^O_2_ (^18^O_2_)	*ν*_O–O_/cm^–1 16^O_2_ (^18^O_2_)	Medium
**Superoxo group**			
C*c*O (beef heart)^*a*^	572 (548)	—	H_2_O, pH 7.4
C*c*O (bovine heart)^*b*^	571 (545)	—	H_2_O, pH 7.2
Fe/Cu[NMePr]^*c*^	570 (544)	—	CH_2_Cl_2_
FeCuArOH^*d*^	575 (549)	—	DMF
[(L^N4-OH^)Cu/Fe(TMPIm)]^*e*^	574 (548)	—	CH_3_CN/THF
FeTPPS/CuTerpyCD_2_^*f*^	578 (551)	—	H_2_O, pH 7.0

**μ-Peroxo group**			
LS-4DCHIm^*g*^	585, 591 (564)	876, 863 (820)	MeTHF
[L^OH^Fe/Cu]^*h*^	—	799 (752)	CH_3_CN/toluene
[(L^N4-OH^)Cu/Fe(TMPIm)]^*e*^	611 (584)	787, 803 (751)	CH_3_CN/THF

^*a*^ Ref. 34.

^*b*^ Ref. 6.

^*c*^ Ref. 14.

^*d*^ Ref. 35.

^*e*^ Ref. 15.

^*f*^This work.

^*g*^ Ref. 37.

^*h*^ Ref. 36.

**Fig. 7:**
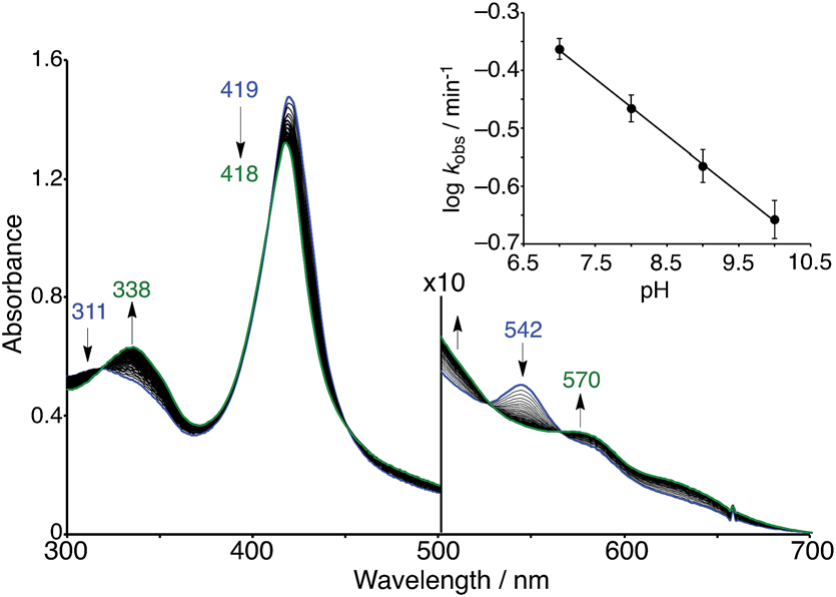
Spontaneous conversion of the superoxo (PFe^III^–O_2_^–^/Cu^I^CD_2_) complex in 0.05 M phosphate buffer at pH 7.0 and 25 °C. The spectrum was recorded at 15 s intervals. The inset shows the logarithmic first-order rate constants (*k*_obs_) for the conversion as a function of the pH of the solution.

The superoxo PFe^III^–O_2_^–^/Cu^I^CD_2_ complex was gradually converted to another state when the solution was allowed to stand at pH 7 and 25 °C under aerobic conditions (Fig. 7). The absorption spectra showed several isosbestic points and the final spectrum (shown as a green line in Fig. 7) was coincident to that of the oxidised Fe^III^TPPS/Cu^II^TerpyCD_2_ complex (Fig. 5). EPR spectral changes also support oxidation of the superoxo PFe^III^–O_2_^–^/Cu^I^CD_2_ species to the Fe^III^TPPS/Cu^II^TerpyCD_2_ complex (Fig. S4‡). The first-order rate constants (*k*_obs_) for the conversion were determined from the absorbance change at various pH conditions. Interestingly, the superoxo complex was more rapidly converted at lower pH (Fig. 7 inset). The linear pH/log *k*_obs_ dependency at pH 7–10 (slope = –0.11) suggests that the conversion is partially accelerated by a proton-coupled process.^39^ Collman *et al.* have reported that the rate of the O_2_ reduction catalysed by their PFe/CuL_*n*_ model complex is pH-dependent and increases at lower pH.^40^ We have previously reported that the autoxidation rate of the O_2_ complex in the PFe^II^/CD dimer system without any distal functions is independent of pH in the neutral pH region (7–10), whereas it is accelerated at pH below 6 and above 10.^24^ Therefore, the pH-rate dependency at the neutral pH region suggests that the water molecules gathered at the distal Cu site promote the conversion of the PFe^III^–O_2_^–^/Cu^I^CD_2_ complex to the oxidised PFe^III^–(OH)–Cu^II^CD_2_ complex.

**Fig. 8:**
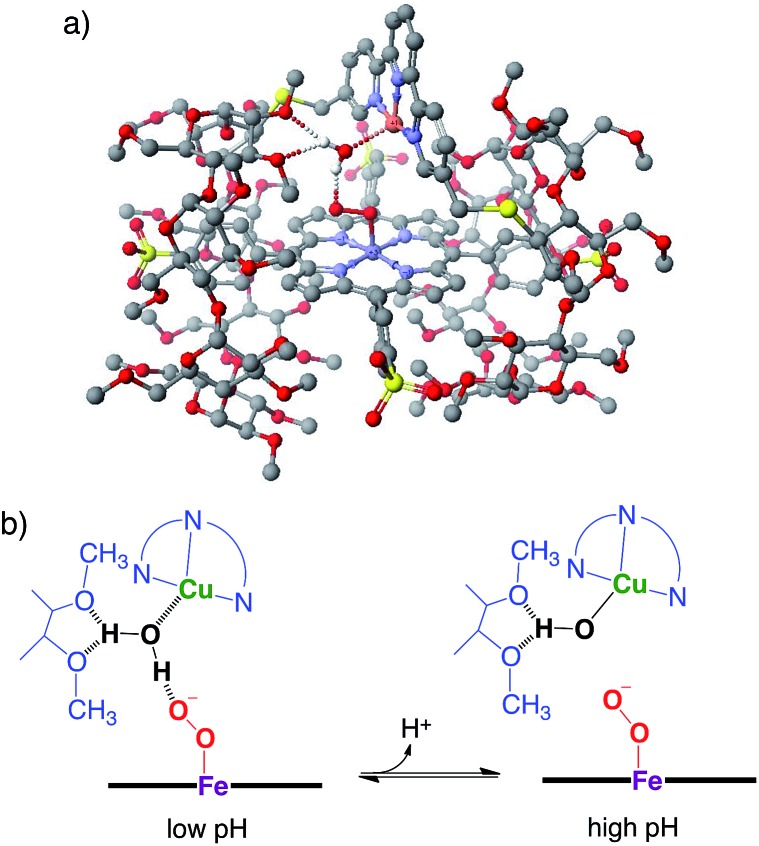
The superoxo PFe^III^–O_2_^–^/Cu^I^CD_2_ complex with a water molecule. (a) The molecular model constructed using CONFLEX/MM3 (extensive search) parameters in Scigress version 2.2.1 software program (Fujitsu). Hydrogen atoms, except for water, are omitted for clarity. (b) The possible acid–base equilibrium of the water, where the proton-donation to the superoxo PFe^III^–O_2_^–^ moiety is likely to occur at low pH.

The quantum chemical study on native C*c*O^8^ proposes that a water molecule coordinating to the distal copper ion facilitates the conversion of compound A to compound P through the formation of the hydroperoxo Fe^III^–OOH intermediate that has not been experimentally detected. Thus, the involvement of a water molecule in the present PFe^III^–O_2_^–^/Cu^I^CD_2_ complex is likely to occur. In addition, molecular modelling suggests that a water molecule bound to the distal copper ion can induce protonation of the superoxo complex (Fig. 8a), where the methoxy groups of the CD dimer are suitable to provide two hydrogen bonding sites to the water. The pH-dependent decomposition of the superoxo complex, as shown in Fig. 7, might be explained by the acid–base equilibrium of the water molecule (Fig. 8b), where the proton-donation to the superoxo complex is likely to induce the O–O bond cleavage as proposed in C*c*O^8^ and/or the proton-assisted autoxidation reaction similar to myoglobin.^41^,^42^

The O_2_ binding in the present complex was practically irreversible; the O_2_ complex of Fe^II^TPPS/Cu^I^TerpyCD_2_ was never converted to its Fe^II^/Cu^I^ deoxy complex, even when the O_2_ complex once formed was dissolved in a deoxygenated buffer (Fig. S5‡). In contrast, the deoxy complex was observed in the Fe^II^TPPS/TerpyCD_2_ complex without copper under the same experimental conditions.^43^ This result indicates that the O_2_ bound to PFe^II^ is tightly held by the distal Cu^I^L_3_ complex, as previously demonstrated by the Fe/Cu superoxo complex.^14^ The tight O_2_ binding was also confirmed by observing ligand exchange with CO. The ligand exchange occurred slowly over ∼30 min when the Fe/Cu superoxo complex was dissolved in a CO saturated buffer (Fig. S5‡), whereas it occurred instantaneously in the absence of distal Cu complex or in the absence of O_2_ (Fig. S5‡). The ligand exchange of O_2_ with CO also rapidly occurs in the previous Fe^II^TPPS/CD dimer systems.^20^,^24^ The significantly slow ligand exchange of PFe^II^–O_2_^–^/Cu^I^L_3_ with CO caused by distal Cu complex might be related to the lower CO/O_2_ affinity ratio of native C*c*O (0.1) in comparison to that of myoglobin (20–50) or haemoglobin (200–250).^44^

### Electrochemical analysis for the O_2_ reduction

**Fig. 9:**
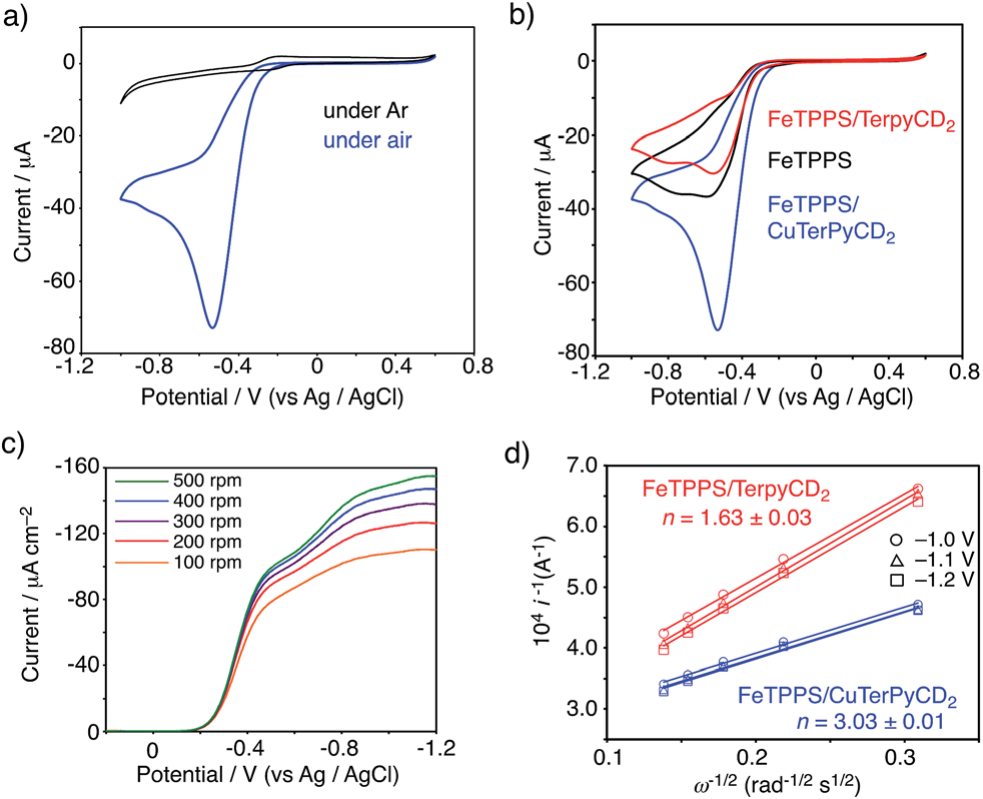
(a, b) CV of the FeTPPS/CuTerpyCD_2_ complex and its reference samples absorbed on the glassy carbon electrode with Nafion (5 wt% dispersion, 10 μL) in pH 7 phosphate buffer at a scan rate of 0.1 V s^–1^ using Ag/AgCl and Pt wire as the reference counter electrodes, respectively. (c) LSV data for the FeTPPS/CuTerpyCD_2_ complex (10 nmol) coated with Nafion (5 wt% dispersion, 10 μL) on a glassy carbon electrode in air saturated pH 7.0 phosphate buffer at a scan rate of 10 mV s^–1^ at multiple rotations using Ag/AgCl and a Pt wire as the reference and counter electrodes, respectively. (d) Koutecky–Levich plots for the FeTPPS/CuTerpyCD_2_ and FeTPPS/TerpyCD_2_ complexes at the potentials of –1.0, –1.1 and –1.2 V to determine the average number of electrons (*n*) used for the O_2_ reduction reaction.

To evaluate the C*c*O-like function of this system, we monitored the electrocatalytic O_2_ reduction reaction.^45^–^47^ The cyclic voltammogram (CV) of the Fe^III^TPPS/Cu^II^TerpyCD_2_ complex immobilized on a glassy carbon electrode showed a reversible redox couple at *E*_1/2_ = –0.21 V (*vs.* Ag/AgCl) in a deoxygenated buffer solution (under Ar, Fig. 9a, black line). The result is similar to those of the previously reported PFe/CuL_*n*_ hetero-binuclear systems; the Fe^III^/Fe^II^ and Cu^II^/Cu^I^ redox waves appear at the same potentials.^31^,^46^ In an air-saturated buffer, the CV of the Fe^III^TPPS/Cu^II^TerpyCD_2_ complex showed a large catalytic current below –0.25 V because of O_2_ reduction (Fig. 9a, blue line). A comparison of the CVs of the Fe^III^TPPS/Cu^II^TerpyCD_2_ complex with those of the reference samples, *i.e.*, Fe^III^TPPS and Fe^III^TPPS/TerpyCD_2_ (Fig. 9b), clearly indicates the effect of the Fe/Cu hetero-binuclear structure in the O_2_ reduction; the Fe^III^TPPS/Cu^II^TerpyCD_2_ complex showed a very large catalytic current starting from a lower onset potential (Δ*E*_onset_ = –40 mV). The O_2_ reduction process was then studied by linear sweep voltammetry (LSV) using a rotating disk electrode (RDE, Fig. 9c). The LSVs of the Fe^III^TPPS/Cu^II^TerpyCD_2_ and Fe^III^TPPS/TerpyCD_2_ complexes showed diffusion limited catalytic O_2_-reduction currents below –1.0 V *vs.* Ag/AgCl. In the case of FeTPPS without the CD dimer, the current was never saturated in LSV due to a slow reaction rate of the iron porphyrin with O_2_ on the disk electrode (Fig. S6‡). The saturated currents observed in the Fe^III^TPPS/Cu^II^TerpyCD_2_ and Fe^III^TPPS/TerpyCD_2_ complexes at various rotation rates were analysed using the Koutecky–Levich equation to determine the average number of electrons (*n*) used in the O_2_ reduction (Fig. 9d).^48^ A significant increase in the *n* value was observed for the Fe/Cu hetero-binuclear complex (*n* = 3.03 ± 0.01) compared to the control sample without copper (*n* = 1.63 ± 0.03).^49^ Therefore, we conclude that the terpyridyl Cu complex associated with FeTPPS in our model system facilitates the catalytic O_2_ reduction as an electron source, as proposed in the mechanism of native C*c*O^3^ and as proven using the synthetic model systems.^5^,^45^,^48^

## Conclusions

In conclusion, we have synthesized a water-soluble biomimetic model complex for the heme *a*_3_/Cu_B_ hetero-binuclear active centre of C*c*O by utilizing a supramolecular complexation, and characterised its reactivity with O_2_. To the best of our knowledge, this is the first example of a totally synthetic C*c*O model that works in a completely aqueous solution. In common with compound A of native C*c*O, we have identified the PFe^III^–O_2_^–^/Cu^I^CD_2_ superoxo complex as the O_2_ adduct in our model system in aqueous solution, whereas the PFe^III^–O_2_–Cu^II^L_*n*_ μ-peroxo complexes tend to form in the other synthetic model systems in anhydrous organic solvents. The pH-dependent conversion of the PFe^III^–O_2_^–^/Cu^I^CD_2_ superoxo complex to its oxidised μ-hydroxo PFe^III^–(OH)–Cu^II^CD_2_ complex suggested the involvement of water molecules in the formation of the superoxo complex in aqueous solution. We believe that our aqueous model system will help to clarify the long-standing arguments with regard to the native and synthetic model systems in C*c*O chemistry.

## Conflicts of interest

The authors declare no conflict of interest.

## Supplementary Material

Supplementary information
