# Supplementary material for: A water-soluble supramolecular complex that mimics the heme/copper hetero-binuclear site of cytochrome c oxidase
Source: Chem Sci. 2018 Jan 15;9(7):1989–95. doi: 10.1039/c7sc04732k (PMC5892347; doi:10.1039/c7sc04732k)
Supplement: Supplementary file 1 [file SC-009-C7SC04732K-s001.pdf]

Electronic Supplementary Material (ESI) for Chemical Science.
This journal is © The Royal Society of Chemistry 2018

Supporting Information for

A Water-Soluble Supramolecular Complex that Mimics the Heme/Copper Hetero-Binuclear Site of Cytochrome c Oxidase

Hiroaki Kitagishi, Daiki Shimoji, Takehiro Ohta, Ryo Kamiya, Yasuhiro Kudo, Akira Onoda, Takashi Hayashi, Jean Weiss, Jennifer A. Wytko and Koji Kano

Contents:

Experimental Section

References in Supporting Information

Figure S1. ^{1}H NMR spectrum of $TerpyCD_2$.

Figure S2. MALDI-TOF mass spectrum of $TerpyCD_2$.

Figure S3. UV-vis spectral change of $Fe^{III}TPPS/Cu^{II}TerpyCD_2$ as a function of pH.

Figure S4. EPR spectra of the $FeTPPS/CuTerpyCD_2$ systems.

Figure S5. Ligand exchange reaction in the O_2 complex of $Fe^{II}TPPS/Cu^{I}TerpyCD_2$.

Figure S6. Linear sweep voltammetry for $Fe^{III}TPPS$ and $Fe^{III}TPPS/TerpyCD_2$.

Experimental section

Instruments. ^{1}H NMR spectra were recorded on a JNM-ECA500 spectrometer (500 MHz). Matrix-assisted laser desorption/ionization time-of-flight (MALDI-TOF) mass spectra were taken on a Bruker Daltonics autoflex speed spectrometer. Direct analysis in real time (DART) mass measurements were carried out using a Shimadzu LCMS 2020 instrument equipped with a DART-SVP ion source. Electrospray ionization time-of-flight (ESI-TOF) mass spectra were taken on a JEOL JMS-T100CS spectrometer. UV-vis spectra were recorded on a Shimadzu MultiSpec-1500 photodiode-array spectrometer with a thermostatic cell holder. Electron paramagnetic resonance (EPR) measurements were made using a JEOL JES-TE200 spectrometer (X-band). Resonance Raman scattering was dispersed by a single polychromator (Ritsu Oyo Kogaku, MC-100DG) with a 405 nm laser (LM-405-PLR-40-2, ONDAX) excitation and was detected by a liquid-nitrogen-cooled CCD detector (HORIBA JOBIN YVON, Symphony 1024 × 128 Cryogenic Front Illuminated CCD Detector. Cyclic voltammetry (CV) were measured using a CompactStat potentiostat (Ivium Tech). Electrochemical analyses using a rotating ring disk electrode (RRDE) were conducted with AFMSRCE rotator (Pine instruments) and an ALS 610B electrochemical analyzer.

Materials. Fe^{III}TPPS was synthesized as previously reported.[1] Synthesis of TerpyCD$_2$ is described below. Other chemical substances were purchased and used as received unless otherwise noted. Phosphate buffer (0.05 M, pH 7.0) was prepared using NaH_2PO_4 and $NaHPO_4$ (Wako). Pure O_2 (99.998%), N_2 (99.998%), and CO (99.9%) gases were purchased from Sumitomo Seika Chemicals. $^{18}O_2$ (99 atom%) was purchased from Isotec, Inc.

5-Methyl-2-trimethylstannylpyridine.[2]

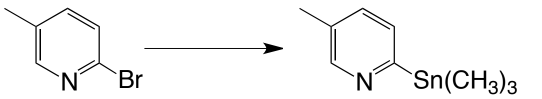

The solution of 5-methyl-2-bromopyridine (2.95 g, 17.2 mmol) in dry diethylether (65 mL) was cooled at –78°C under an Ar atmosphere. To the solution was slowly added *n*-BuLi (1.6 M in hexane, 11.3 mL, 18.5 mmol). After 2 h, to the solution was added dropwise the solution of trimethyltin chloride (4.3 g, 21.6 mmol) in dry diethyl ether (25 mL). The solution was stirred overnight at room temperature. To the solution was added saturated aqueous ammonium chloride solution (10 mL). The organic layer was washed with the saturated aqueous ammonium chloride solutions (30 mL × 3), dried over Na_2SO_4, and evaporated. The residue was dissolved in hexane and insoluble materials were removed. The solvent was evaporated to give 5-methyl-2-trimethystannylpyridine (4.32 g, 98%): ^{1}H NMR (500 Mz, $CDCl_3$) δ 8.59 (*s*, 1H), 7.33 (*m*, 2H), 2.29 (*s*, 3H), 0.32 (*s*, 9H, J_{SnH} = 27 Hz); MS (DART) *m*/*z* 258 $(M + H)^+$.

5,5''-Dimethyl-2,2':6'2''-terpyridine[3]

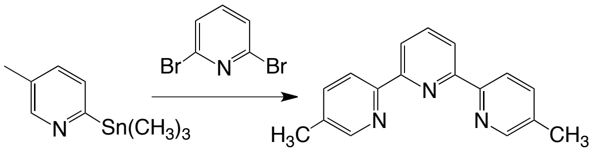

5-Methyl-2-trimethylstannylpyridine (823 mg, 3.2 mmol) and 2,6-dibromopyridine (255 mg, 1.1 mmol) were dissolved in toluene (30 mL) and the solution was bubbled with Ar for 15 min. To the solution was added $Pd(PPh_3)_4$ (76 mg, 0.065 mmol) and the solution was further bubbled with Ar for additional 15 min. The solution was refluxed for 4 days under Ar atmosphere. After the solution was cooled to room temperature, the solvent was removed by evaporation. The residue was dissolved in 6 M HCl (20 mL) and washed with dichloromethane (30 mL). The organic phase was extracted with 6 M HCl (20 mL × 3). The combined aqueous solution was neutralized with 25% aqueous NH_4OH to pH 9. The resulting solid was collected and dissolved in dichloromethane. After the solution was dried with Na_2SO_4, the solvent was removed in vacuo. The solid was recrystallized from ethyl acetate to give 5,5''-dimethyl-2,2':6'2''-terpyridine (0.29 g, 68%): ^{1}H NMR (500 Mz, $CDCl_3$) δ 8.50 (*s*, 1H), 8.49 (*d*, 2H, *J* = 8.4 Hz), 8.38 (*d*, 2H, *J* = 7.8 Hz), 7.91 (*t*, 1H, *J* = 7.48 Hz), 7.63 (*d*, 2H, *J* = 8.4 Hz), 2.39 (*s*, 6H); MS (DART) *m*/*z* 262 $(M + H)^+$.

5,5”-Bis(bromomethyl)-2,2’:6’2”-terpyridine[4]

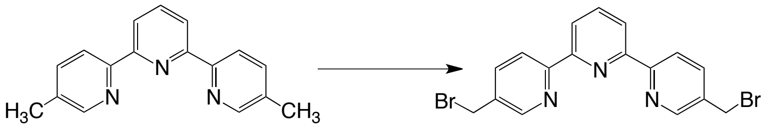

5,5”-Dimethyl-2,2’:6’2”-terpyridine (500 mg, 1.92 mmol), *N*-bromosuccinimide (1.71 g, 9.59 mmol) and AIBN (52 mg, 0.31 mmol) were dissolved in tetrachloromethane (40 mL). The solution was refluxed for 32 min. The insoluble material was filtered and the solid was washed well with tetrachloromethane. The filtrate solution was combined and evaporated. The residue was dissolved in dichloromethane (30 mL) and the solution was washed with 0.5 M aqueous $Na_2S_2O_3$ (30 mL × 2). The aqueous phase was extracted with dichloromethane (50 mL). The combined organic phase was dried with Na_2SO_4 and evaporated. The residue was dissolved in minimum amount of dichloromethane and the solution was cooled at 4°C for 16 h. The resulting solid was collected to give 5,5”-bis(bromomethyl)-2,2’:6’2”-terpyridine (0.16 g, 20%); ^{1}H NMR (500 Mz, $CDCl_3$) δ 8.70 (*d*, 2H, *J* = 2.3 Hz), 8.60 (*d*, 2H, *J* = 8.0 Hz), 8.46 (*d*, 2H, *J* = 8.3 Hz), 7.98 (*t*, 1H, *J* = 8.3 Hz), 7.90 (*dd*, 2H, *J* = 8.0, 2.3 Hz), 4.57 (*s*, 4H); MS (DART) *m*/*z* 418 $(M + H)^+$.

5,5”-Bis(mercaptomethyl)-2,2’:6’2”-terpyridine

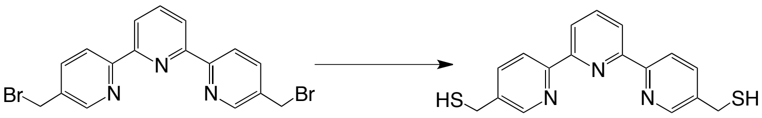

5,5”-Bis(bromomethyl)-2,2’:6’2”-terpyridine (120 mg, 0.29 mmol) and thiourea (49 mg, 0.64 mmol) were dissolved in dry ethanol (13 mL). The solution was then refluxed for 2.5 h. To the solution was added 5 M aqueous NaOH (5 mL) and the mixture was further refluxed for 1.5 h. After the solution was cooled to room temperature, the solution was poured into 1 M HCl (25 mL) and then pH of the solution was adjusted to approximately 6.5 with 0.1 M aqueous NaOH. The suspended solution was extracted with dichloromethane (20 mL × 3). The organic layer was dried with Na_2SO_4 and evaporated to give 5,5”-bis(mercaptomethyl)-2,2’:6’2”-terpyridine; ^{1}H NMR (500 Mz, $CDCl_3$) δ 8.69 (*d*, 2H, *J* = 2.3 Hz), 8.59 (*d*, 2H, *J* = 8.6 Hz), 8.42 (*d*, 2H, *J* = 7.5 Hz), 8.10 (*t*, 1H, *J* = 7.5 Hz), 8.00 (*dd*, 2H, *J* = 8.6, 2.3 Hz), 3.87 (*d*, 4H, *J* = 8.0 Hz), 3.16 (*t*, 2H, *J* = 8.0 Hz); MS (DART) *m*/*z* 326 $(M + H)^+$. This compound was air-sensitive and immediately used for the next reaction.

TerpyCD$_2$

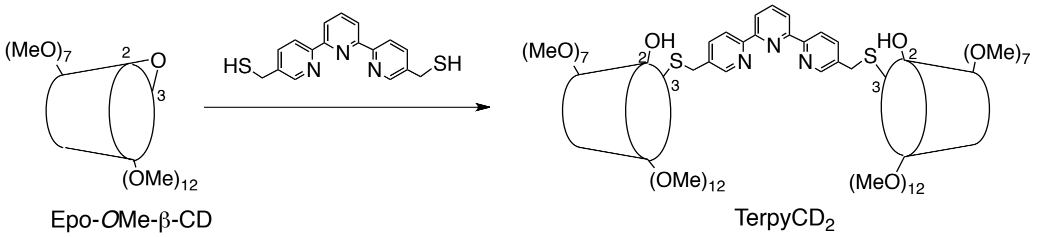

2,3-Monoepoxy-per-*O*-methylated β-CD (Epo-*O*Me-β-CD, 542 mg, 0.39 mmol)[5] was dissolved in Ar-saturated 0.1 M aqueous $NaHCO_3$ (40 mL). To the solution was added a suspended solution of 5,5”-bis(mercaptomethyl)-2,2’:6’2”-terpyridine (82 mg, 0.25 mmol) in methanol (5 mL). The solution was refluxed under an Ar atmosphere for 18 h. After the solution was cooled to room temperature, the solution was evaporated to remove methanol from the solution. The resulting aqueous solution was extracted with chloroform (30 mL × 5). The organic layers were combined, dried with Na_2SO_4, and evaporated to dryness. The residue was purified with silica gel column chromatography with a gradient elution from chloroform/acetone (5/2, v/v) to chloroform, and then to chloroform/methanol (25/1, v/v). The product was further purified by an HPLC system (Jpn. Anal. Ind., Tokyo, Japan) equipped with a GS310 GPC column (eluent, chloroform/methanol = 1/1) to afford TerpyCD$_2$ (0.21 g, 36%); ^{1}H NMR (500 Mz, $CDCl_3$) δ 8.70 (*s*, 2H), 8.56 (*d*, 2H, *J* = 8.0 Hz), 8.42 (*d*, 2H, *J* = 8.0 Hz), 7.94 (*m*, 3H), 5.32–4.64 (*m*, 14H), 4.32–3.05 (*m*, 200H), 3.16 (*t*, 2H, *J* = 8.0 Hz); MS (MALDI-TOF) *m*/*z* 3113.2 $(M + Na)^+$; Anal. Calcd. for $C_{139}H_{227}N_3O_{68}S_2{\bullet}3H_2O{\bullet}CHCl_3$: C, 51.49; H, 7.22; N, 1.29. Found: C, 51.34; H, 7.11; N 1.39%. ^{1}H NMR and MALDI-TOF mass spectra are shown in Figures S1 and S2.

Sample preparation for the O_2 complex of Fe^{II}TPPS/Cu^{I}TerpyCD$_2$. The ferrous/cuprous complex was prepared by adding $Na_2S_2O_4$ to a mixture of Fe^{III}TPPS and Cu^{II}TerpyCD$_2$ (one equiv to the porphyrin unit) in 0.05 M phosphate buffer at pH 7.0. The redox potential of dithionite is negative (E = –0.66 V vs SHE)[6] enough to reduce both Fe^{III} and Cu^{II} to Fe^{II} and Cu^{I} oxidation states, and excessively added dithionite completely decomposed O_2 in the aqueous solution.[7] Excess $Na_2S_2O_4$ and its oxidized derivatives were removed by a Sephadex G-25 desalting column. During column treatment, the Fe^{II}TPPS/Cu^{I}TerpyCD$_2$ complex became O_2 complex by capturing atmospheric O_2. To avoid decomposition of the O_2 complex, all the above procedures were conducted at 4°C, and the resulting stock solution was placed in a refrigerator at 4°C.

Sample preparation for resonance Raman measurements. The solution of the O_2 complex was prepared as described above (the concentration of the O_2 complex was approximately 0.1 mM) and the solution in an NMR tube was immediately frozen by liquid N_2. In case of the $^{18}O_2$ sample, the atmosphere in the NMR tube was replaced by $^{18}O_2$ gas before transferring the aqueous solution of the Fe^{II}TPPS/Cu^{I}TerpyCD$_2$ complex. The spectrum was recorded in Dewar condenser filled with the liquid N_2.

Electrochemical measurements.[8,9] For cyclic voltammetry (CV) measurements, the samples (Fe^{III}TPPS, Fe^{III}TPPS/TerpyCD$_2$, and Fe^{III}TPPS/Cu^{I}TerpyCD$_2$) in water (1 mM) were dropped onto the polished glassy carbon electrode (3 mm in diameter, BAS) and dried at room temperature. The complex on the electrode was immobilized with Nafion® solution (5 wt%, 10 μL, Sigma-Aldrich) and dried again at room temperature. The modified electrode was rinsed well with water and transferred to an electrochemical cell containing 0.05 M phosphate buffer (pH 7). CV was measured in the potential range of +0.6 to –1.0 V (vs Ag/AgCl reference electrode) at a scan rate of 100 mV/s. For linear sweep voltammetry (LSV) measurements, the modified glassy carbon electrode (5 mm in diameter) with sample/Nafion® was fixed to an RRDE system (E6 series Change Disk tips, Pine Instruments). The scans were made at a rate of 10 mV/s in the potential range of +0.3 to –1.2 V.

References in Supporting Information

1) K. Kano, H. Kitagishi, S. Tamura, A. Yamada, *J. Am. Chem. Soc.* **2004**, *126*, 15202–15210.

2) P. F. H. Schwab, F. Fleischer, J. Michl, *J. Org. Chem.* **2002**, *67*, 443–449.

3) U. S. Schubert, C. Eschbaumer, G. Hochwimmer, *Synthesis* **1999**, *5*, 779–782.

4) S. Köytepe, S. Erdoğan, T. Seçkin, *J. Hazard Mater.* **2009**, *162*, 695–702.

5) K. Kano, H. Kitagishi, M. Kodera, S. Hirota, *Angew. Chem. Int. Ed.* **2005**, *44*, 435–438. 2005

6) G. M. Stephen, *Eur. J. Biochem.* **1978**, *85*, 535–547.

7) Z. Tao, J. Goodisman, A.-K. Souid, *J. Phys. Chem. A* **2008**, *112*, 1511–1518.

8) A. Onoda, N. Inoue, S. Campidelli, T. Hayashi, *RSC Adv.* **2016**, *6*, 65936–65940.

9) H. Harada, A. Onoda, T. Uematsu, S. Kuwabata, T. Hayashi, *Langmuir* **2016**, *32*, 6459–6467.

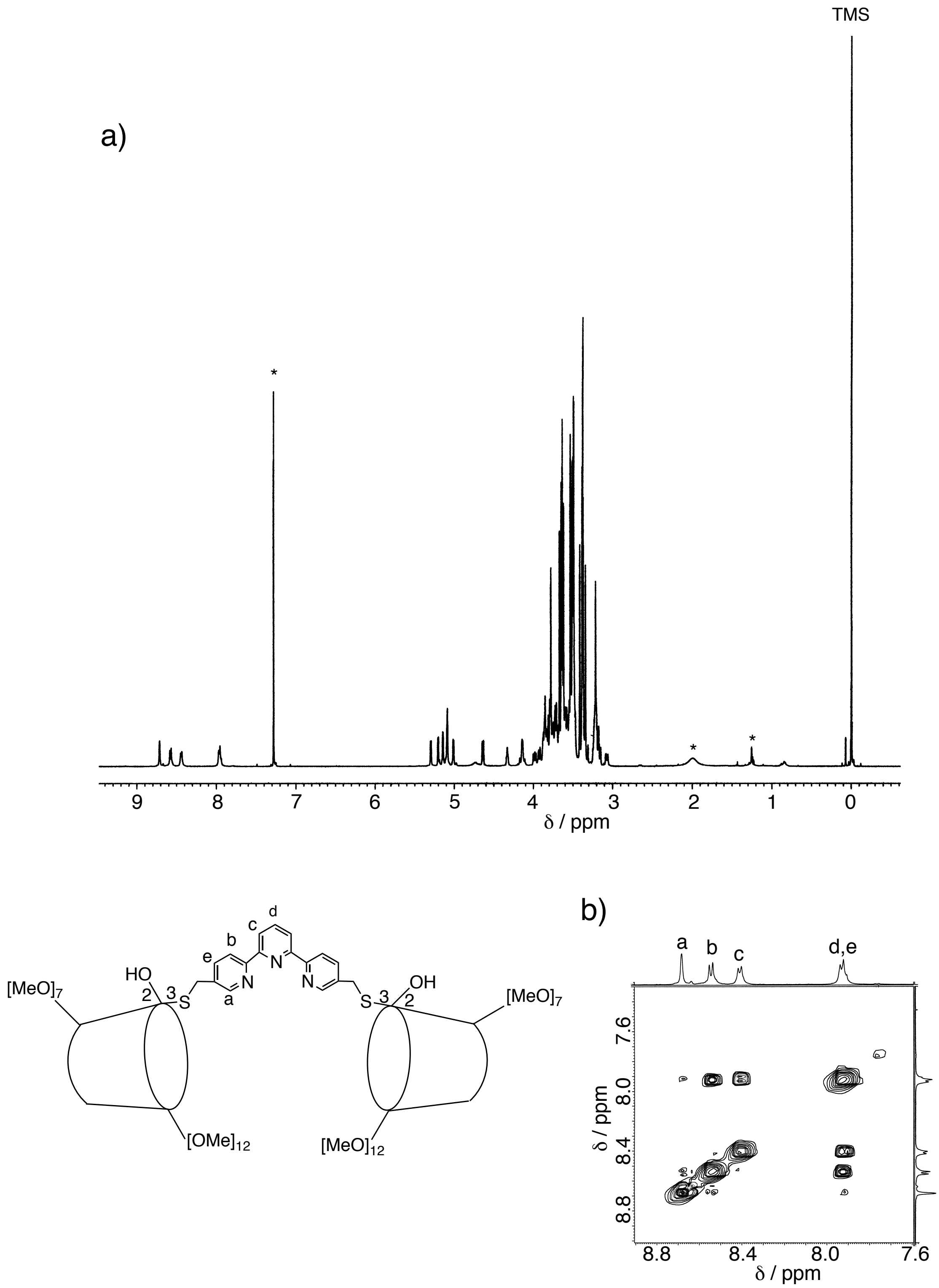

Figure S1. ^{1}H NMR spectrum of TerpyCD$_2$ in CDCl$_3$ (500 MHz, standard; TMS). (a) 1D ^{1}H NMR spectrum. Asterisks (*) denote the residual solvent peaks. (b) ^{1}H–^{1}H COSY spectrum for the assignment of the aromatic signals of TerpyCD$_2$.

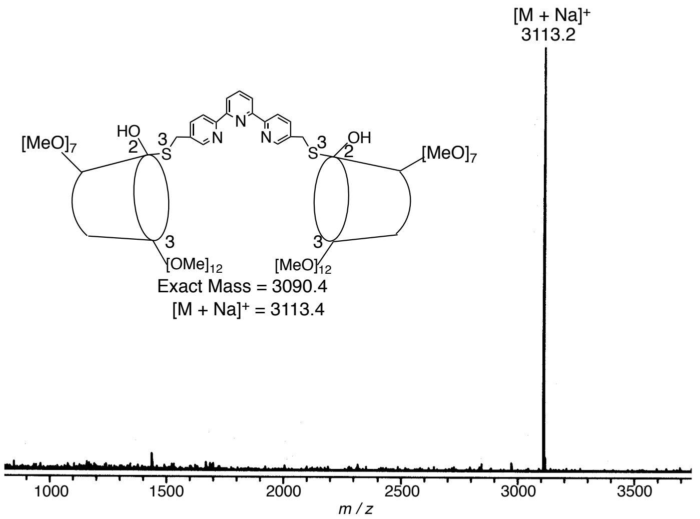

Figure S2. MALDI-TOF mass spectrum of $TerpyCD_2$ with a subsequent addition of α-cyano-4-hydroxycinnamic acid matrix (positive mode).

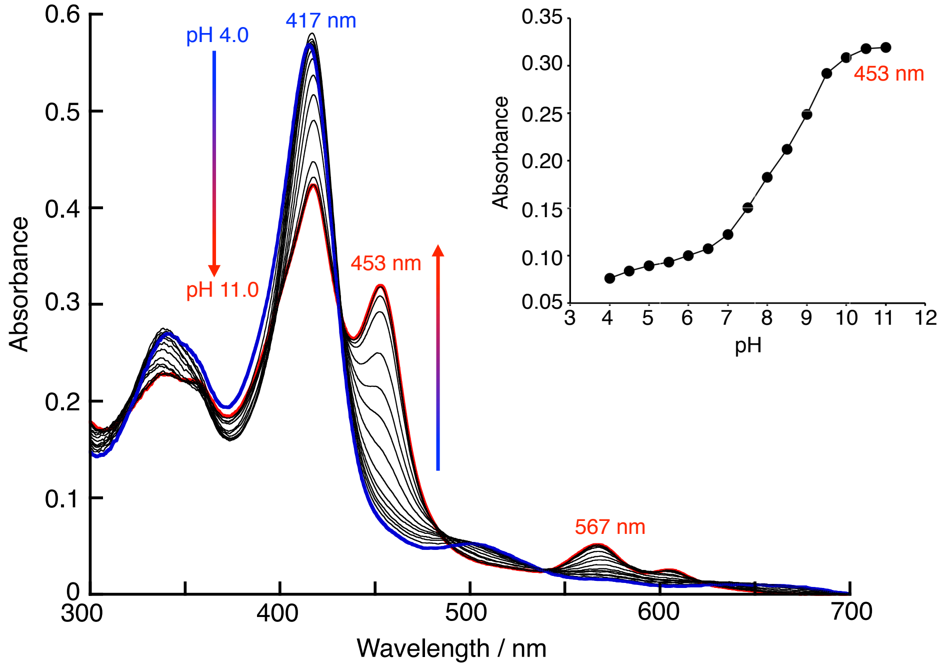

Figure S3. UV-vis spectral change of the $Fe^{III}TPPS/Cu^{II}TerpyCD_2$ complex (4 μM) in aqueous 0.1 M $NaClO_4$ solution at 25°C as a function of pH of the solution (The pH was adjusted by $HClO_4$ and NaOH). Inset shows the plot of the absorbance at 453 nm as a function of pH. From the pH titration curve, the pK_a value for the acid-base equilibrium of $[PFe^{III}–O–Cu^{II}CD_2]^{3-}$ and $[PFe^{III}–(OH)–Cu^{II}CD_2]^{2-}$ was determined to be 8.8. The pK_a value was consistent with the predicted value by Karlin and Blackburn (pK_a ~ 8).

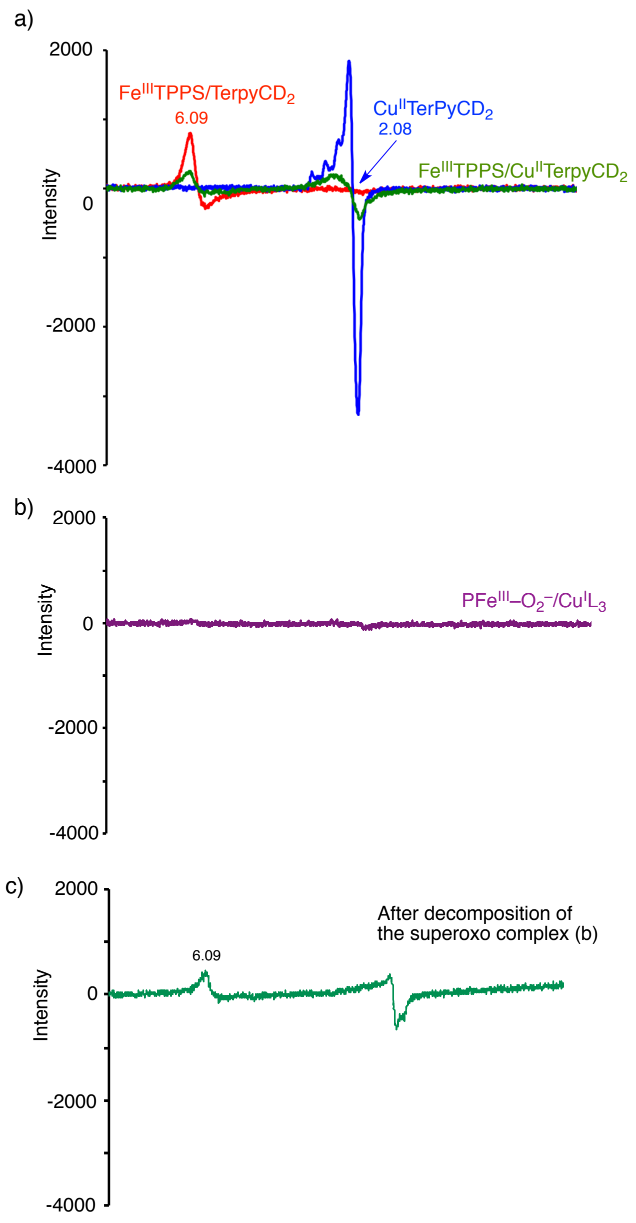

Figure S4. EPR spectroscopic analysis. (a) Overlaid EPR spectra of the frozen aqueous solutions of the Fe^{III}TPPS/TerpyCD$_2$, Cu^{II}TerpyCD$_2$, and Fe^{III}TPPS/Cu^{II}TerpyCD$_2$ complexes (1 mM each). The weakened signals at around g = 6 and g = 2 in the Fe^{III}TPPS/Cu^{II}TerpyCD$_2$ complex is ascribed to the antiferromagnetic coupling between the proximity of Fe^{III} and Cu^{II} metal ions. (b,c) EPR spectra of the frozen aqueous solution of the O_2 complex of Fe^{II}TPPS/Cu^{I}TerpyCD$_2$ (*ca*. 1 mM) before (b) and after autoxidation (c). Conditions: temperature, 77 K; microwave frequency, 9.4 GHz; microwave power, 1.2 mW; modulation frequency, 100 kHz; modulation width, 0.32 mT; time constant, 0.03 sec.

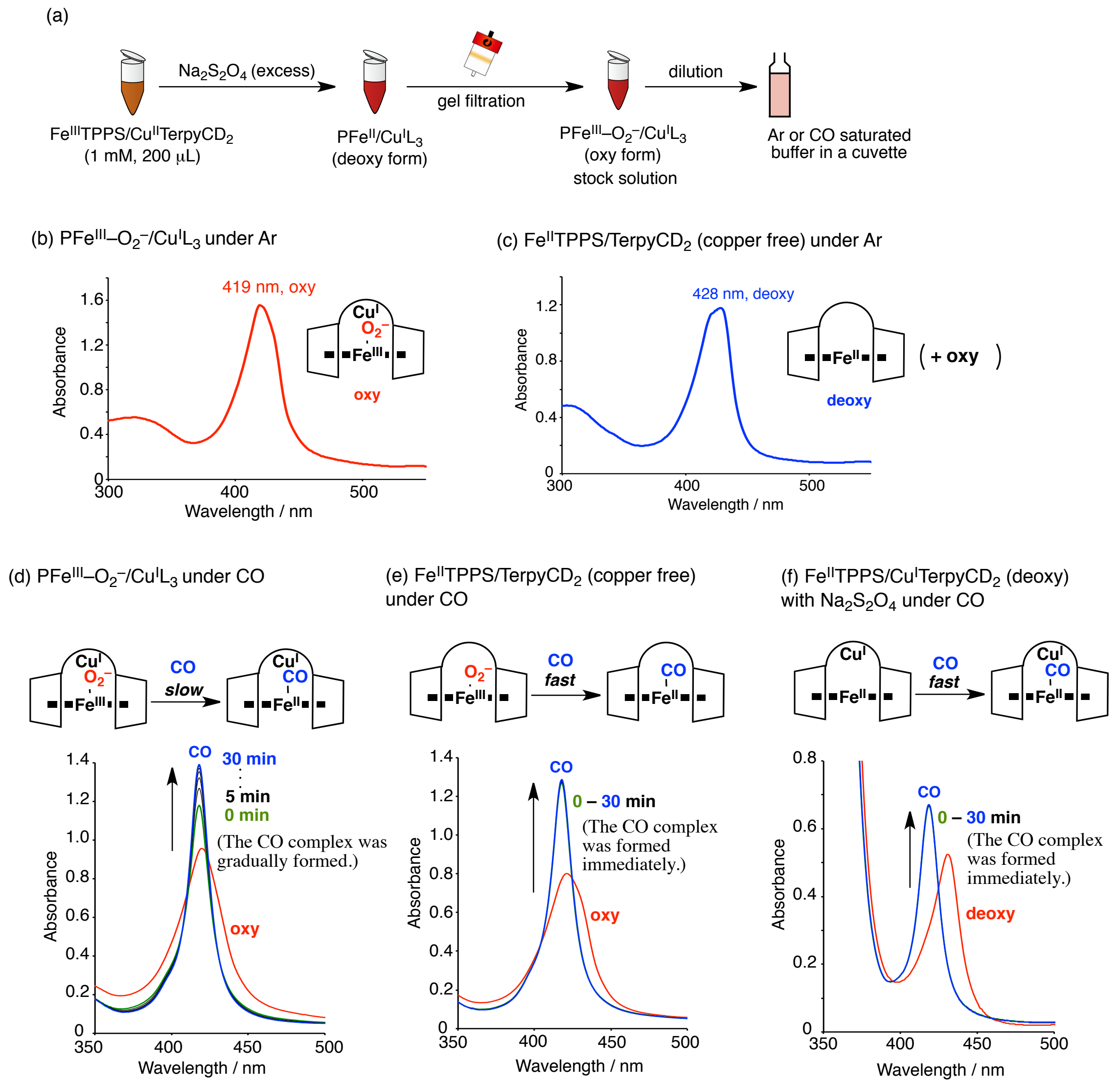

Figure S5. Ligand exchange reaction of the Fe^{II}TPPS/Cu^{I}TerpyCD$_2$ systems. (a) The experimental procedure. (b, c) UV-vis spectra of the complexes (PFe^{III}–O_2^-/Cu^{I}CD$_2$ and its copper-free complex) dissolved in the Ar-saturated buffer solution. The oxy complex was observed in the Fe^{II}TPPS/Cu^{I}TerpyCD$_2$ complex under an Ar atmosphere (b), while the deoxy complex was partly observed at 428 nm in the Cu-free complex (c). (d–f) The time-course UV-vis spectral changes of the complexes (PFe^{III}–O_2^-/Cu^{I}CD$_2$ and its copper-free complex) after being dissolved in the CO-saturated buffer without (d, e) and with excess $Na_2S_2O_4$ (f). The spectra were recorded at 5 min intervals after the dilutions of the complex in the CO saturated buffer. The ligand exchange occurred slowly over ~30 min when the Fe/Cu superoxo complex was dissolved in the CO saturated buffer (d), whereas it occurred instantaneously in the absence of distal Cu complex (e) or in the absence of O_2 (with excess dithionite, f). These experiments demonstrated that the

$PFe^{II}/Cu^{I}CD_2$ hetero-binuclear complex held O_2 very tightly and the deoxygenation and the ligand exchange with CO were unlikely to occur when the O_2 complex once formed in the $PFe^{II}/Cu^{I}CD_2$ hetero-binuclear system.

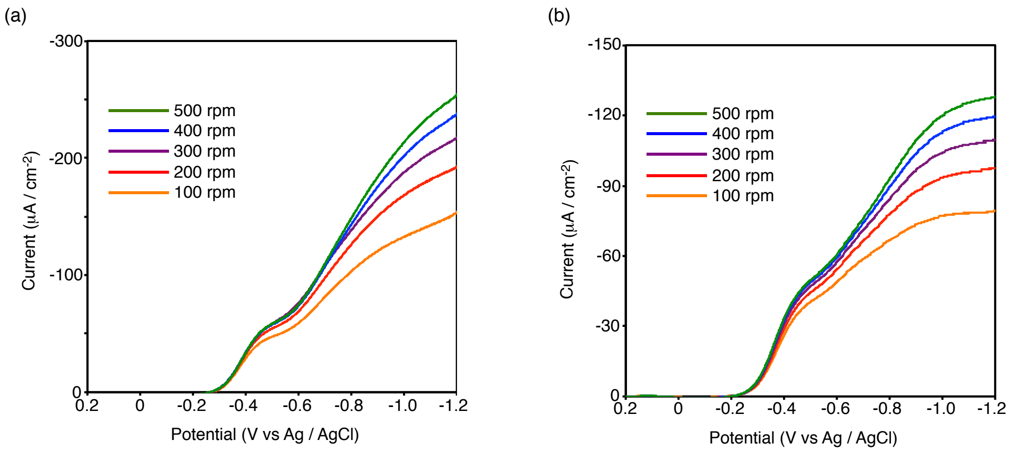

Figure S6. Linear sweep voltammetry for Fe^{III}TPPS (a) and Fe^{III}TPPS/TerpyCD$_2$ (b). These samples (10 nmol) coated with Nafion 5 wt% dispersion (10 μL) on a glassy carbon electrode in air saturated pH 7.0 phosphate buffer at a scan rate of 10 mV/s at multiple rotations using Ag/AgCl as reference and Pt wire as counter electrodes. In the case of Fe^{III}TPPS, the current was not saturated in LSV due to its slow reaction rate with O_2 on the disk electrode.
